# The first complete mitochondrial genomes of sawtail surgeonfishes (Acanthuridae: *Prionurus*)

**DOI:** 10.1080/23802359.2019.1699465

**Published:** 2019-12-12

**Authors:** William B. Ludt, Luiz A. Rocha, Prosanta Chakrabarty

**Affiliations:** a Department of Ichthyology, Natural History Museum of Los Angeles County, Los Angeles, CA, USA;; b Section of Ichthyology, California Academy of Sciences, San Francisco, CA, USA;; c Ichthyology Section, Museum of Natural Science, Department of Biological Sciences, Louisiana State University, Baton Rouge, LA, USA

**Keywords:** Mitochondrion, Acanthuriformes, reef fish, herbivore, phylogenetics

## Abstract

Surgeonfishes of the family Acanthuridae are primarily large-bodied herbivores that provide critical ecosystem services to coral reefs. Five out of the six genera that comprise the family have had mitochondrial genomes sequenced, with the exception of the genus *Prionurus*. Here, for the first time, we assemble and annotate the mitochondrial genomes of two sawtail surgeonfishes. The circular genomes of *P. biafraensis* and *P. laticlavius* are 16,552 bp and 16,531 bp in length, respectively, and contain 13 protein-coding genes, 22 transfer RNA genes, 2 ribosomal RNA genes, and a control region. Gene arrangement and codon usage were similar to reported mitochondrial genomes of other surgeonfish genera, and a phylogenetic analysis of protein-coding genes recovers a topology for Acanthuridae that is consistent with nuclear analyses.

Surgeonfishes (Acanthuriformes: Acanthuridae) are large-bodied coral reef fishes that occur in all tropical and some sub-tropical seas (Randall 2001). The family comprises six genera and 85 recognized species (Fricke et al. 2019), most of which are herbivores that provide important ecosystem functions to reefs and an important link between primary producers and higher trophic levels (Randall 2001; Marshell and Mumby 2015). Mitochondrial genomes for most surgeonfish genera have been sequenced (Yamanoue et al. 2007; Devadhasan et al. 2016a, 2016b; Huang et al. 2017), with the exception of the genus *Prionurus*. Here, we report whole mitochondrial genomes for *P. biafraensis* (Blache and Rossignol 1961) and *P. laticlavius* (Valenciennes 1846) for the first time.

Specimens of *P. biafraensis* were collected in 2006 from Ilhéu de Santana, São Tomé and Príncipe (0.2417°N, 6.7586°W; tissue CAS-PBI01), and *P. laticlavius* was collected from the Pacific coast of Costa Rica in 2014 (10.5806°N, 85.6762°W; voucher LSUMZ 17879, tissue LSUMZ-F 6789). Mitochondrial genomes were recovered from off-target sequencing reads of a hybrid target capture study of ultraconserved elements from Ludt et al. (2019). Raw sequences from that study had sample-specific dual-indexing barcodes and low-quality bases removed using trimmomatic (Bolger et al. 2014) as part of the illumiprocessor wrapper (Faircloth 2013). Sequences were then mapped to a reference mitochondrial genome of *Naso lopezi* (GenBank accession NC_009853.1) using the Geneious mapping algorithm in Geneious Prime 2019.2.3. An annotated consensus sequence was then created with a minimum 5X base coverage. Mitochondrial genomes of *Prionurus* were aligned with genomes from all other surgeonfish genera, as well as two outgroup taxa from the Acanthuriformes, and protein-coding genes (PCGs) were extracted for phylogenetic analysis. IQtree v1.6.12 (Nguyen et al. 2015) was used to determine the most appropriate partitioning scheme and substitution models (Chernomor et al. 2016; Kalyaanamoorthy et al. 2017), and a maximum likelihood analysis was conducted with 1000 ultrafast bootstrap replicates (Hoang et al. 2018) (Figure 1).

**Figure 1.**
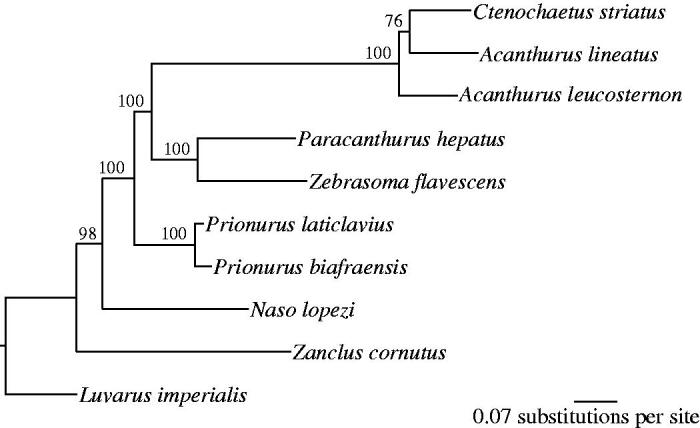
Phylogenetic relationship of 10 acanthuriform fishes constructed with a partitioned maximum likelihood approach using 13 PCGs found in the mitochondrial genome. Numbers at nodes represent bootstrap values. GenBank accession numbers: *Ctenochaetus striatus* (KU244260.1), *Acanthurus lineatus* (NC_010108.2), *A. leucosternon* (EU136032.1), *Paracanthurus hepatus* (NC_029237.1), *Zebrasoma flavescens* (NC_009874.1), *Prionurus laticlavius* (MN703418), *P. biafraensis* (MN703417), *Naso lopezi* (NC_009853.1), *Zanclus cornutus* (NC_009852.1), *Luvarus imperialis* (NC_009851.1).

Mitochondrial genomes for both species of *Prionurus* contained 13 PCGs, 22 tRNA genes, 2 rRNA genes, and a control region. The genome for *P. biafraensis* (GenBank accession MN703417) was 16,552 bp long, had a mean coverage of 25.2X, and had a GC content of 44.6%, while the genome for *P. laticlavius* (GenBank accession MN703418) was 16,531 bp long, had a mean coverage of 101X, and a GC content of 44.2%. The arrangement of genes was identical to other surgeonfish genera, and there was a total of 64 bp of intergenic spacer sequences across 11 regions ranging from 1 to 38 bp. The start codon ATG was the most common, occurring in all PCGs except for COXI and ND6 which had the start codons GTG and TAC, respectively. The stop codon TAA terminated six PCGs (ND1, COXI, ATP8, ND4L, ND5, and ND6) and incomplete termination codons of T–– or TA– were found in seven PCGs (ND2, COX2, ATP6, COX3, ND3, ND4, and CYTB). The recovered maximum likelihood topology (Figure 1) is strongly supported and matches those recovered by previous studies including nuclear markers (Sorenson et al. 2013).
